# Effect of Pneumococcal Conjugate Vaccine on Pneumonia Incidence Rates among Children 2–59 Months of Age, Mongolia, 2015–2021

**DOI:** 10.3201/eid3003.230864

**Published:** 2024-03

**Authors:** Claire von Mollendorf, Munkhchuluun Ulziibayar, Cattram D. Nguyen, Purevsuren Batsaikhan, Bujinlkham Suuri, Dashtseren Luvsantseren, Dorj Narangerel, John de Campo, Margaret de Campo, Bilegtsaikhan Tsolmon, Sodbayar Demberelsuren, Eileen M. Dunne, Catherine Satzke, Tuya Mungun, E. Kim Mulholland

**Affiliations:** Murdoch Children’s Research Institute, Melbourne, Victoria, Australia (C. von Mollendorf, C.D. Nguyen, J. de Campo, M. de Campo, E.M. Dunne, C. Satzke, E.K. Mulholland);; The University of Melbourne, Melbourne (C. von Mollendorf, C.D. Nguyen, J. de Campo, M. de Campo, E.M. Dunne, C. Satzke, E.K. Mulholland);; National Center for Communicable Diseases, Ulaanbaatar, Mongolia (M. Ulziibayar, P. Batsaikhan, B. Suuri, D. Luvsantseren, B. Tsolmon, T. Mungun);; Ministry of Health, Ulaanbaatar (D. Narangerel);; Mongolian National University of Medical Sciences, Ulaanbaatar (B. Tsolmon);; World Health Organization, Ulaanbaatar (S. Demberelsuren);; Peter Doherty Institute for Infection and Immunity, Melbourne (C. Satzke);; London School of Hygiene and Tropical Medicine, London, UK (E.K. Mulholland)

**Keywords:** pneumonia, pneumococcal conjugate vaccine, PCV13, hospitalization, children, bacteria, Mongolia, respiratory infections

## Abstract

Starting in June 2016, the 13-valent pneumococcal conjugate vaccine (PCV13) was introduced into the routine immunization program of Mongolia by using a 2+1 dosing schedule, phased by district. We used prospective hospital surveillance to evaluate the vaccine’s effect on pneumonia incidence rates among children 2–59 months of age over a 6-year period. Of 17,607 children with pneumonia, overall adjusted incidence rate ratios showed decreased primary endpoint pneumonia, very severe pneumonia, and probable pneumococcal pneumonia until June 2021. Results excluding and including the COVID-19 pandemic period were similar. Pneumonia declined in 3 districts that introduced PCV13 with catch-up campaigns but not in the 1 district that did not. After PCV13 introduction, vaccine-type pneumococcal carriage prevalence decreased by 44% and nonvaccine-type carriage increased by 49%. After PCV13 introduction in Mongolia, the incidence of more specific pneumonia endpoints declined in children 2–59 months of age; additional benefits were conferred by catch-up campaigns.

Globally, the most common infectious cause of death among children 1–59 months of age is lower respiratory tract infection (*1*). Despite vaccine availability, *Streptococcus pneumoniae* causes a substantial proportion of severe pneumonia cases, attributed to 18.3% of severe pneumonia episodes and 32.7% of all pneumonia deaths in children globally (*2*). Pneumonia disease burden is highest among younger children and in certain regions such as southern Asia and Africa (*2*).

Mongolia is a lower-middle-income country in central Asia. Half of the Mongolia population of 3.3 million live in the capital city of Ulaanbaatar (*3*). Similar to other low- and middle-income countries (LMICs), several demographic and socioeconomic factors in Mongolia increase the risk for childhood pneumonia (*4*). Rapid urbanization with expansion of informal living areas and coal use during winter has resulted in poor air quality in Ulaanbaatar (*5*). Air pollution exacerbates respiratory diseases such as asthma and increases the risk for pneumonia (*6*).

In the past 2 decades, pneumococcal conjugate vaccines (PCVs) have had a substantial public health effect globally; effectiveness against hospitalization for invasive pneumococcal disease, clinical pneumonia, and radiologically confirmed pneumonia has been demonstrated (*7*,*8*). Modeling has estimated that, in children <5 years of age, introduction of 13-valent PCV (PCV13) resulted in a reduction of 175 million cases of pneumococcal disease and 625,000 associated deaths worldwide over 10 years (*9*). Among those cases, 14 million illnesses and 374,550 deaths resulted from pneumococcal pneumonia (*9*); however, 6 countries in Asia have yet to introduce PCV into their national immunization programs, and in 2021, >25 million children in those regions still did not have access to the vaccines (*10*). Data from Asia with regard to pneumonia burden and PCV effect are lacking; only 2 studies have demonstrated the effect of PCV13 (*11*,*12*).

Starting in 2016, PCV13 was introduced into the routine infant immunization program of Mongolia, phased by district, in the context of an expanded pneumonia surveillance program to monitor vaccine effect (*13*). Baseline data estimated that clinical pneumonia incidence among children 2–59 months was 31.8 cases/1,000 population and for severe pneumonia was 19.2 cases/1,000 population (*14*). To ensure sustainability of the program in Mongolia, PCV13 was introduced in stages because the country was transitioning from Gavi funding (*15*). 

Our study goal was to estimate the effect of PCV13 introduction on clinical and radiologic pneumonia endpoints among hospitalized children 2–59 months of age living in 4 districts of Ulaanbaatar, Mongolia, over a 6-year period. The study was approved by the Medical Ethics Review Committee at the Mongolian Ministry of Health and the Royal Children’s Hospital Human Research Ethics Committee (HREC 33203). Written informed consent was obtained from all parents/caregivers for enrolled children before any study procedures were conducted.

## Methods

### Study Setting

Expanded hospital-based pneumonia surveillance was initiated in 4 districts of Ulaanbaatar in April 2015 as previously described (*13*,*14*). Mongolia introduced PCV13 into the national immunization program in a 2+1 schedule (2, 4, and 9 months) by district: June 2016 (Songinokhairkhan [SKD] and Sukhbaatar [SBD]), July 2017 (Bayanzurkh [BZD]), and March 2018 (Chingeltei [CHD]). Catch-up campaigns were instituted in the districts in which PCV13 was introduced in 2016 and 2017 (*13*,*14*). During 2017–2021, PCV13 coverage among the target age group from all introduced districts was reported to be 95%–98% (*16*).

### Study Population and Design

During April 2015–June 2021, we enrolled children 2–59 months of age who were admitted to 1 of 4 four participating district hospitals (or the tertiary hospital if they resided in one of the relevant districts) and met the specific study case definition for clinical pneumonia. We excluded patients with bronchiolitis and bronchitis. Protocol details have been previously published (*13*) (Appendix). Blood samples, nasopharyngeal swab samples, and chest radiographs were collected for all enrolled patients or for whom consent was provided. To ensure that no eligible patients were missed, dedicated study staff ensured that patients were correctly enrolled by clinical hospital staff.

The primary study outcome was World Health Organization (WHO)–defined primary endpoint pneumonia (PEP) (*17*). Secondary outcomes were clinical pneumonia (all cases); severe pneumonia (WHO 2005 case definition [*18*]); very severe pneumonia (severe cases complicated by empyema, intensive care unit admission, persistent severe disease after discharge, hypoxia, or death [*14*]); hypoxic pneumonia (oxygen saturation <90%); probable pneumococcal pneumonia (PPP) (*19*) (elevated C-reactive protein with either PEP [*19*] or high pneumococcal nasopharyngeal carriage); or definite pneumococcal pneumonia (positive blood or pleural fluid culture) and pneumococcal carriage (*13*).

### Sample Collection and Laboratory Procedures

We adhered to WHO recommended methods for nasopharyngeal sample collection, handling, and transport (*20*). We tested nasopharyngeal swab samples for pneumococci by using *lytA* real-time quantitative PCR and molecular serotyping by DNA microarray (Appendix) (*21*). We tested 1,000 patients/year for pneumococci, including all patients with PEP (primary objective) and a random sample of remaining patients.

### Statistical Analyses

We summarized categorical variables with frequency counts and percentages and demographic variables by district and overall. To determine changes before and after PCV13 introduction, we compared characteristics of children during the 2 periods. We calculated crude annual incidence rates for April–March because surveillance started in April 2015 and pneumonia was highly seasonal and most cases were identified during winter. We obtained annual population estimates for denominators from the Mongolian Ministry of Health. We calculated CIs for incidence estimates by using a Poisson distribution. We based the definitions of pre-PCV13 and post-PCV13 periods on month of vaccine introduction at the district level. We calculated crude incidence rates and incidence rate ratios (IRRs) comparing pre-PCV13 and post-PCV13 periods for all patients and stratified them by district and age group.

We calculated adjusted IRRs (aIRRs) for different pneumonia endpoints comparing pre-PCV13 and post-PCV13 periods by using negative binomial regression with separate models for data until February 2020 (excluding the COVID-19 pandemic period) and June 2021 (end of study). All models included terms for PCV13 introduction, district, age group, and a categorical variable for each calendar month elapsed (to account for secular trends), with log-transformed population denominators included as an offset. To allow for a differential effect between districts, we included an interaction term between PCV13 and district for district-specific effects. The model coefficients were exponentiated to obtain IRRs with 95% CIs. We calculated percent reduction in pneumonia rates as (1 – IRR) × 100%. We conducted 2 sensitivity analyses for IRR calculations. We first introduced a 1-year lag period for effect of PCV introduction and then stratified IRRs by age group (2–23 months and 24–59 months).

We used univariable and multivariable log-binomial regression to estimate crude and adjusted prevalence ratios (aPR) for overall, PCV13-type and non-PCV13–type prevalence of pneumococcal carriage. To adjust prevalence ratios, we used a common set of confounders, selected by using a directed acyclic graph based on current literature (Appendix Figure 1). We calculated prevalence ratios by comparing the post-PCV13 with the pre-PCV13 period for all endpoints. Reductions in PCV13 carriage were calculated as (1 – aPR) × 100%. We used Stata statistical software 17.0 (StataCorp LLC, https://www.stata.com) to analyze data.

## Results

During April 1, 2015–June 30, 2021, a total of 55,691 children 2–59 months of age with acute lower respiratory tract infections were admitted to one of the study hospitals; 17,688 (32%) were assessed according to the study case definition, received study consent, and were enrolled (Appendix Figure 2). Among the 17,607 confirmed to meet all study eligibility criteria, 71% were 2–23 months of age, 54% were male and 46% female, and most were admitted during autumn and winter (Appendix Table 1). More than two thirds of households had single children <5 years of age, and 21% of children attended kindergarten. Most participants (15,248 [87%]) had a risk-factor questionnaire completed by a parent or caregiver; 81% (14,184), underwent chest radiography; and 87% (15,411) had nasopharyngeal swab samples collected and processed, of which 6,545 swabs were tested for pneumococci. Of 13,602 children for whom complete data were available to assess PPP, 11% met the case definition. Blood cultures were performed for 15,232 (87%) children, but only 14 (0.1%) were culture-positive for *S. pneumoniae*. For 2 children, *S. pneumoniae* was cultured from pleural fluid; and for 1 child, blood culture was also positive.

The highest numbers of patients were enrolled from the largest districts, SKD and BZD. Differences were observed between the 4 study districts (Appendix Table 1). Most households in CHD (2,984/3,703 [81%]) and SKD (3,259/4,568 [71%]) used coal or wood as the main fuel source, and only half of the households in SBD and BZD used those smoky fuels. The highest proportions of participants living in crowded households were in CHD (32%) and SKD (36%) or living in informal housing were also in those same 2 districts (39% for CHD and 45% for SKD). Overall, 77% of participants had severe pneumonia; proportions were slightly higher in CHD (79%) and SKD (81%). A total of 37% of participants had very severe pneumonia; percentages were highest in BZD (43%) and CHD (46%). Of 13,755 children with interpretable chest radiographs, 1,813 (13%) had PEP (Appendix Table 1).

**Figure 1:**
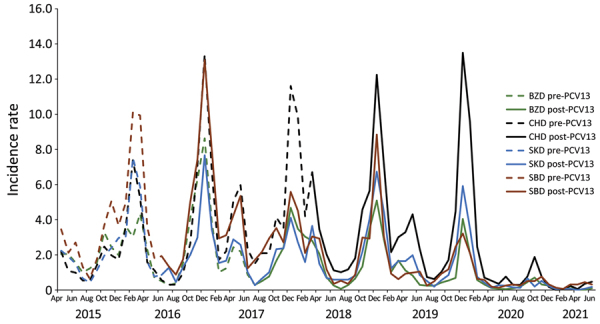
All clinical pneumonia incidence rates (cases/1,000 population) by month and district in children 2–59 months of age, Ulaanbaatar, Mongolia, April 2015–June 2021. BZD, Bayanzurkh District; CHD, Chingeltei District; PCV13, 13-valent pneumococcal conjugate vaccine; SBD, Sukhbaatar District; SKD, Songinokhairkhan District.

Pneumonia incidence rates were highly seasonal; case numbers were highest during winter (October–February) (Figure 1; Appendix Figure 3). After PCV13 introduction, peak incidence of all clinical pneumonia decreased, except in CHD, which had no PCV catch-up campaign (Figure 1). Pneumonia incidence decreased from February 2020 through June 2021, when COVID-19 restrictions, including kindergarten/school closures, were in place. No winter peak was observed during the 2020–21 season (Figure 1; Appendix Figure 3). Overall, 32% of admitted patients met the study case definition, which was intended to exclude patients with milder pneumonia (Appendix Figure 4).

The profile of participants differed before and after introduction of PCV13 (Appendix Table 2). Compared with the pre-PCV13 period, percentages were lower for children previously admitted (48% before vs. 42% after; p<0.0001), with hypoxia (22% before vs. 17% after; p<0.0001), or with primary endpoint pneumonia (14% before vs. 13% after; p = 0.007) in the post-PCV13 period. The percentage of children with severe and very severe pneumonia in the post-PCV13 period was also reduced (Appendix Table 2).

By March 2020 (early COVID-19 pandemic restrictions), changes for crude IRRs varied by pneumonia diagnosis and district (Appendix Table 3). For all districts combined, IRR was reduced for all patients with all clinical pneumonia (21%, 95% CI 18%–23%), PEP (20%, 95% CI 12%–27%), severe pneumonia (23%, 95% CI 20%–25%), very severe pneumonia (26%, 95% CI 22%–29%), hypoxic pneumonia (34%, 95% CI 29%–39%), and PPP (38%, 95% CI 31%–44%). Individual districts mainly showed reductions, except for CHD, which showed increases in IRRs in cases of all clinical, severe, and very severe pneumonia. By March 2021, which included a period of COVID-19 restrictions, additional reductions were observed in line with reduced case numbers, and PEP was reduced by 36% (95% CI 29%–42%) (Appendix Table 3). We found some variability by age group; slightly larger reductions were observed for the 24–59-month age group compared with the younger age group (Appendix Table 4). Annual incidence rates were highest in 2016 in SKD, SBD, and BZD, but CHD showed high incidence rates until 2019 (Appendix Table 5).

**Figure 2:**
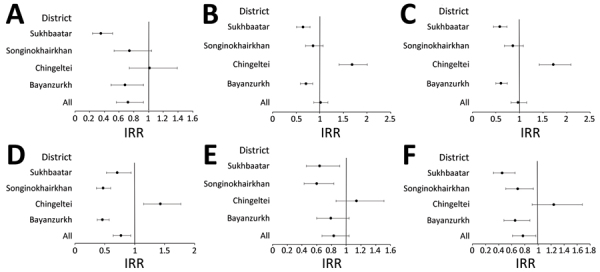
Adjusted IRRs for pneumonia endpoints for pre-vaccine period (April 2015–February 2020, excluding COVID-19 pandemic period) in study of effect of pneumococcal conjugate vaccine on pneumonia incidence rates among children 2–59 months of age, Mongolia, 2015–2021. A) Primary endpoint pneumonia; B) all pneumonia; C) severe pneumonia; D) very severe pneumonia; E) hypoxic pneumonia; F) probable pneumococcal pneumonia. Error bars indicate 95% CIs. IRR, incidence rate ratio.

**Figure 3:**
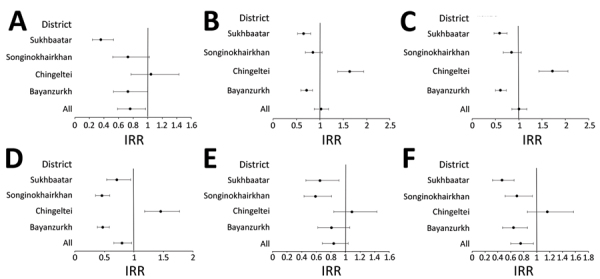
Adjusted IRRs for pneumonia endpoints post-vaccine period (April 2015–June 2021, including COVID-19 pandemic period) in study of effect of pneumococcal conjugate vaccine on pneumonia incidence rates among children 2–59 months of age, Mongolia, 2015–2021. A) Primary endpoint pneumonia; B) all pneumonia; C) severe pneumonia; D) very severe pneumonia; E) hypoxic pneumonia; F) probable pneumococcal pneumonia. Error bars indicate 95% CIs. IRR, incidence rate ratio.

To account for secular trends and district effect not accounted for in crude IRRs, we calculated aIRRs for different pneumonia endpoints until February 2020 before extensive COVID-19 lockdown measures (Figure 2; Appendix Table 6). Those aIRRs showed a reduction in all clinical pneumonia rates in 3 of the districts (BZD 0.71, 95% CI 0.59–0.85; SKD 0.86, 95% CI 0.70–1.07; SBD 0.64, 95% CI 0.51–0.79) and an increase in 1 district (CHD 1.68, 95% CI 1.41–2.01) where PCV13 was introduced last without a catch-up campaign. The trends observed in the other pneumonia endpoints were similar across districts. For all districts combined by February 2020, aIRRs showed a reduction in PEP (0.72, 95% CI 0.56–0.93), very severe pneumonia (0.77, 95% CI 0.64–0.93), and PPP (0.77, 95% CI 0.61–0.97); however, reductions were not shown for severe pneumonia (0.97, 95% CI 0.82–1.15), hypoxic pneumonia (0.83, 95% CI 0.67–1.04), or all clinical pneumonia (1.01, 95% CI 0.87–1.17) (Figure 2; Appendix Table 6). Reductions were similar until June 2021 (Figure 3, Appendix Table 6).

A total of 6,545 samples were tested for pneumococci. Overall, 3,056 (47%) were positive for pneumococcal carriage and 2,557 (84%) were culturable and had serotyping results, of which 1,058 (41%) had PCV13-type serotypes, 1,267 (50%) had non–PCV13-type serotypes, and 232 (9%) had both types of serotype identified. In all districts combined, overall pneumococcal carriage prevalence (any serotype) did not change between the pre-PCV13 (48%) and post-PCV13 (46%) periods (adjusted prevalence ratio [aPR] 0.98, 95% CI 0.92–1.04) overall or in the individual districts (Table). PCV13-type carriage overall was reduced by 44% (aPR 0.56, 95% CI 0.51–0.62) and in each district ranging from 41% in BZD and SBD to 50% in SKD. Non–PCV13-type carriage increased overall (aPR 1.49, 95% CI 1.32–1.67) and significantly in 2 districts (Table).

**Table:** Carriage prevalence and prevalence ratios for pneumococcal carriage among 6,545 children with pneumonia before and after PCV13 availability, 4 districts, Mongolia, 2015–2021*

Pneumococcal type	Pre-PCV13, no./total	Pre-PCV13 prevalence, % (95% CI)	Post-PCV13 no./total	Post-PCV13 prevalence, % (95% CI)	Unadjusted prevalence ratio (95% CI)	Adjusted prevalence ratio (95% CI)†
Overall pneumococci						
All districts	882/1,837	48.0 (45.7–50.3)	2,174/4,708	46.2 (44.7–47.6)	0.96 (0.91–1.02)	0.98 (0.92–1.04)
Bayanzurkh	263/657	40.0 (36.2–43.9)	363/905	40.1 (36.9–43.4)	1.00 (0.89–1.13)	1.06 (0.93–1.21)
Chingeltei	341/592	57.6 (53.5–61.6)	565/1,194	47.3 (44.4–50.2)	0.82 (0.75–0.90)	0.81 (0.73–0.90)
Songinokhairkhan	184/368	50.0 (44.8–55.2)	953/1,891	50.4 (48.1–52.7)	1.01 (0.90–1.13)	1.00 (0.89–1.12)
Sukhbaatar	94/220	42.7 (36.1–49.5)	293/718	40.8 (37.2–44.5)	0.95 (0.80–1.14)	0.95 (0.79–1.14)
PCV13 serotypes						
All districts	548/1,742	31.4 (29.3–33.7)	742/4,304	17.2 (16.1–18.4)	0.55 (0.50–0.60)	0.56 (0.51–0.62)
Bayanzurkh	161/614	26.2 (22.8–29.9)	119/830	14.3 (12.0–16.9)	0.55 (0.44–0.68)	0.59 (0.47–0.75)
Chingeltei	200/566	35.3 (31.4–39.4)	205/1,077	19.0 (16.7–21.5)	0.54 (0.46–0.64)	0.53 (0.44–0.63)
Songinokhairkhan	127/354	35.9 (30.9–41.1)	306/1,737	17.6 (15.8–19.5)	0.49 (0.41–0.58)	0.50 (0.42–0.61)
Sukhbaatar	60/208	28.8 (22.8–35.5)	112/660	17.0 (14.2–20.0)	0.59 (0.45–0.77)	0.59 (0.44–0.78)
Non-PCV13 serotypes						
All districts	329/1,742	18.9 (17.1–20.8)	1,170/4,304	27.2 (25.8–28.5)	1.44 (1.29–1.60)	1.49 (1.32–1.67)
Bayanzurkh	76/614	12.4 (9.9–15.2)	193/830	23.2 (20.4–26.3)	1.88 (1.47–2.40)	1.95 (1.49–2.55)
Chingeltei	152/566	26.8 (23.2–30.7)	286/1,077	26.5 (23.9–29.3)	0.99 (0.83–1.17)	0.96 (0.79–1.17)
Songinokhairkhan	69/354	19.5 (15.5–24.0)	550/1,737	31.7 (29.5–33.9)	1.62 (1.30–2.03)	1.57 (1.24–1.99)
Sukhbaatar	32/208	15.4 (10.8–21.0)	141/660	21.4 (18.3–24.7)	1.39 (0.98–1.97)	1.26 (0.88–1.81)

*Overall, PCV13 serotypes and non-PCV13 serotypes. PCV13, 13-valent pneumococcal conjugate vaccine. †Adjusted by using a common set of confounders: age, informal housing, other children <5 y of age in the home, coal used for fuel, household income, crowding, maternal education, season, and antimicrobial drug receipt 48 h before admission.

### Sensitivity Trends 

We calculated aIRRs, assuming a delay of 1 year for the effect of PCV13 introduction among all children 2–59 months of age (Appendix Table 7). Results for PEP were similar to those of the main analysis (26% [95% CI 4%–43%] reduction). We observed a greater reduction in clinical pneumonia (24%, 95% CI 9%–36%), severe pneumonia (24%, 95% CI 8%–38%), and very severe pneumonia (30%, 95% CI 14%–44%) compared with the main analyses.

Stratification by age group (2–23 months and 24–59 months) demonstrated a greater reduction in most endpoints among older children. All clinical pneumonia cases were reduced by 12% (95% CI −7% to 27%) (negative numbers indicate an increase), PEP a 38% (95% CI 10%–57%) reduction, severe pneumonia a 13% (95% CI −9% to 30%) reduction, very severe pneumonia a 39% (95% CI 21%–52%) reduction, and hypoxic pneumonia a 31% (95% CI 7%–48%) reduction in all districts combined (Appendix Table 7).

## Discussion

In our large-scale surveillance study in Mongolia, a country with a high burden of respiratory disease, we demonstrated the effect of PCV13 introduction on children hospitalized for pneumonia. We found that phased introduction of PCV13 in 4 districts of Ulaanbaatar resulted in reduced disease incidence, with some variability by district, age, and pneumonia endpoint used. Overall, PCV13 led to similar reductions in cases of PEP (28%), very severe pneumonia (23%), and PPP (23%) but no significant reduction of all clinical pneumonia or severe pneumonia. Reductions were observed in 3 districts in which catch-up campaigns were conducted at the time of vaccine introduction. PCV13-type pneumococcal carriage declined overall (44%) and in each individual district. Non–PCV13-type carriage increased overall and significantly in 2 districts. Our surveillance program is one of few programs reporting PCV13 effect on pneumonia for a high-burden LMIC in Asia.

Many countries have used invasive pneumococcal disease (IPD) to determine PCV effect. Because IPD is rare and requires robust laboratory capacity, using IPD is often not possible in LMICs, nor is it an ideal metric in countries such as Mongolia with small populations and few annual IPD cases detected. Pneumonia surveillance can be an indicator of PCV effect. A challenge in studying PCV effect on pneumonia is that young children do not produce sputum, very few cases are bacteremic, and no diagnostic tests are available for nonbacteremic pneumococcal pneumonia in this age group.

In Fiji, a time-series analysis 5 years after PCV10 introduction found a reduction in pediatric hospitalizations for pneumonia, varying by age and pneumonia endpoint (*22*). Similar to the Fiji study, we found that compared with younger children, the reduction of pneumonia was greater among children 24–59 months of age, although a lower proportion of children in that group were fully vaccinated. It is likely that a higher percentage of cases in the older group were caused by pneumococcus and in the younger (<2 years of age) group by respiratory syncytial virus (*23*).

A recent systematic review found a decline in pneumonia hospitalization incidence among children after PCV introduction, although the magnitude of the decline across different endpoints and settings displayed heterogeneity (*24*). The review demonstrated that PCV effect tended to increase as the pneumonia outcome increased in diagnostic specificity for pneumococcal disease (*24*). We observed substantial declines in carriage of PCV13 serotypes as well as declines in pneumonia outcomes considered more likely to be caused by pneumococcus, such as PEP and very severe pneumonia.

The decrease in pneumonia cases during 2020 and 2021 probably results from measures put in place to combat the COVID-19 pandemic. Mongolia instituted kindergarten/school closures from the end of January 2020 until September 2021, except for a brief period during late 2020 (*25*,*26*). In addition, travel bans, multiple hard lockdowns, and other public health nonpharmaceutical interventions were instituted (*25*,*27*), and COVID-19 vaccines were available starting in February 2021 (*27*). Studies from other countries have shown that restrictions instituted during the COVID-19 pandemic reduced childhood infections (*28*,*29*).

The use of catch-up campaigns has been encouraged by WHO as a strategy to increase herd immunity (*30*). Observational data from LMICs documenting the effect of catch-up campaigns is limited. A transmission dynamic model using data from Kenya indicated that a catch-up campaign among children <5 years of age prevented additional IPD cases and used fewer doses per case averted than routine introduction only (*31*). In our surveillance program, PCV introduction included a catch-up campaign in 3 of the 4 study districts. Pneumonia incidence was not significantly reduced in the district without catch-up (CHD) but was reduced, especially for more severe pneumonia endpoints, in the other districts. Of note, CHD was the last district to introduce PCV13, and no significant increase in non–PCV13-type carriage was demonstrated. The average annual coverage in eligible age groups in CHD was similar to routine coverage in BZD, where PCV13 was introduced in 2017.

In addition to catch-up campaigns, other explanations for different results between districts are variable smoke exposure, levels of poverty, housing type, crowding, and other factors reflective of known risk factors for pneumonia (*4*). Movement between districts and migration may also have varied over the study period. A previous publication from Mongolia found evidence of direct and indirect vaccine effects on carriage, which varied by formal and informal living conditions (*32*). We observed a reduction (46%) in vaccine-type pneumococcal carriage 3–5 years after introduction in 4 districts. We identified residual circulation of vaccine serotypes (17%) despite high PCV coverage, similar to findings in Malawi and South Africa (*33*,*34*).

One study strength is establishment of an expanded active pneumonia surveillance program on pre-existing WHO invasive bacterial disease surveillance in 4 districts of Ulaanbaatar. All patients admitted for pneumonia were screened daily by clinical staff, and they were enrolled if they met a prespecified case definition. The case definition selected for more severe cases. To ensure that all eligible patients were identified, dedicated study staff monitored weekly enrollments performed by clinical staff. Any eligible patients that were missed were enrolled retrospectively, ensuring a high inclusion rate. The 6-year study included a considerable number of patients admitted for respiratory conditions. A structured questionnaire was completed for participants, and most underwent chest radiography and specimen collection. The radiographs were reread by 2 experienced independent radiologists using WHO guidelines (*17*), and sensitive molecular methods were used to measure pneumococcal carriage and determine serotypes (*20*). In Mongolia, hospitalization is free for all children <5 years of age, which reduces bias associated with access to care. In addition, Mongolia has a structured public healthcare system in which most patients flow from primary care to district hospitals, enabling population-based estimates. The adherence of patients to this referral pathway can sometimes vary, however, by socioeconomic status and setting (*35*).

The first limitation our study was that although we had only 1 year of pre-PCV13 data in all districts, because of a phased PCV13 introduction, we had 2–3 years of data before vaccine introduction in half of the districts. Second, the study included only 4 Ulaanbaatar districts, so the results may not be generalizable to all children in Mongolia, although the included districts are the largest in Ulaanbaatar and half the country’s population live in this city. Third, we did not collect data for a nonrespiratory control condition and could not account for other interventions, such as air pollution measures, which may have affected pneumonia trends. Fourth, the COVID-19 pandemic affected case numbers; however, adjusted IRRs were similar before or including this period. Last, ongoing internal migration of inhabitants and a possible increase in unregistered migrants during a migration ban (2017–2020) (*36*) may have potentially affected denominators and thus incidence rates. In addition, urban redevelopment of traditional tented housing (ger) districts resulted in the temporary relocation of inhabitants from ger to other subdistricts (*37*). Redevelopment and relocation were reported in the ger subdistricts of CHD during 2016 and 2017 (*37*), which may have resulted in lower case numbers reported in these years, because of patients accessing alternative district hospitals, and contributed to an overall rate increase.

In conclusion, PCV13 introduction into the childhood immunization schedule in Mongolia, with catch-up vaccination in 3 districts, resulted in substantially reduced pneumonia incidence. The decreases were more prominent for more severe disease endpoints and in PCV13-type pneumococcal colonization. Other countries that have satisfactory PCV coverage can expect decreased severe pneumonia cases and vaccine-type carriage after vaccine introduction. Countries should consider offering catch-up vaccination when introducing PCV and should monitor changes in disease burden and pneumococcal serotypes through surveillance. Our study adds to limited data available on PCV effects for Asia and for countries transitioning from Gavi financial support.

Appendix Additional information for study of effect of pneumococcal conjugate vaccine on pneumonia incidence rates among children 2–59 months of age, Mongolia, 2015–2021.
