# Supplementary material for: Effect of Pneumococcal Conjugate Vaccine on Pneumonia Incidence Rates among Children 2–59 Months of Age, Mongolia, 2015–2021
Source: Emerg Infect Dis. 2024 Mar;30(3):490–8. doi: 10.3201/eid3003.230864 (PMC10902538; doi:10.3201/eid3003.230864)
Supplement: Appendix — Additional information for study of effect of pneumococcal conjugate vaccine on pneumonia incidence rates among children 2–59 months of age, Mongolia, 2015–2021. [file 23-0864-Techapp-s1.pdf]

Article DOI: https://doi.org/10.3201/eid3003.230864

EID cannot ensure accessibility for supplementary materials supplied by authors. Readers who have difficulty accessing supplementary content should contact the authors for assistance.

Effect of Pneumococcal Conjugate Vaccine on Pneumonia Incidence Rates among Children 2–59 Months of Age, Mongolia, 2015–2021

Appendix

Supplemental Methods

Study setting

Catch-up campaigns were only instituted during the first two years of PCV13 introduction into Songinokhairkhan, Sukhbaatar and Bayanzurkh. In 2018 vaccine was introduced into Chingeltei (in conjunction with the five remaining districts of Ulaanbaatar) without a catch-up campaign as introduction was self-funded by the government.

Study population and design

Of the nine districts of Ulaanbaatar, four (Songinokhairkhan, Sukhbaatar, Chingeltei and Bayanzurkh) were identified by the Government of Mongolia for initial 13-valent pneumococcal conjugate vaccine (PCV13) introduction as part of the country's phased introduction plan. Pneumonia surveillance was also enhanced (from the routine WHO surveillance) in these four districts to evaluate PCV13 impact. The four districts included the two largest districts in Ulaanbaatar and two central districts, making up ~70% of the city's population (*1*).

Children aged 2–59 months admitted with clinical pneumonia, who met the study case definition, were enrolled from April 2015 to June 2021. The all clinical pneumonia case definition included children with cough or difficulty breathing, and respiratory rate ≥50 bpm (for all age groups), or oxygen saturation <90% or a clinical diagnosis of severe pneumonia. Children admitted at one of the four participating secondary district hospitals, or the tertiary hospital if they resided in one of the relevant districts, were included. One of the included district hospitals

(Bayanzurkh) was privatised (*2*). Other private hospitals were not included in the surveillance programme as nearly all children are treated in the public sector for pneumonia. Hospitalisations for children are fully funded by the government (*2*,*3*).

Two standardised questionnaires collected information on demographic variables, presenting symptoms and signs, previous medication, immunisation history, treatment received, and risk factors. Blood samples, nasopharyngeal swabs and chest x-rays were collected for all enrolled cases who consented. Dedicated study staff monitored patient enrolment by clinical hospital staff to ensure that no eligible patients were missed. Participants who were missed by clinical staff were enrolled retrospectively. If participants were enrolled more than 72 hours after admission nasopharyngeal swabs were not collected (*1*).

Case definitions and study outcomes

The enrolment case definition for clinical pneumonia and the specific pneumonia endpoints (study outcomes) are detailed below:

1) All clinical pneumonia surveillance case definition (*1*)

Cough or difficulty breathing, with one of the following:

• an elevated respiratory rate (≥50 bpm for all ages)

• oxygen saturation <90%

• a clinical diagnosis of severe pneumonia

2) WHO-defined primary endpoint pneumonia (*4*):

• End-point consolidation (dense or fluffy opacity that occupies a portion or whole of a lobe or the entire lung that may or may not contain air bronchograms) OR

• Pleural effusion that is in the lateral pleural space and associated with pulmonary parenchymal infiltrate or if the effusion obliterated enough of the hemithorax to obscure an opacity.

3) Severe pneumonia (IMCI 2005 criteria (*5*))

Cough or difficulty breathing and tachypnoea PLUS

• Lower chest indrawing OR

• General danger sign (inability to breastfeed or drink, persistent vomiting, lethargy or reduced level of consciousness, convulsions or severe malnutrition) OR

• Oxygen saturation < 90% or central cyanosis

4) Very severe pneumonia (*6*):

Severe pneumonia with one or more of the following:

• ICU admission/supplementary oxygen

• hypoxia (Oxygen saturation < 90%)

• death

• persistent signs of severe illness post-discharge

• empyema

5) Probable pneumococcal pneumonia (*1*)

Elevated C-reactive protein with

• primary endpoint pneumonia (*7*) OR

• high pneumococcal nasopharyngeal carriage (either high density carriage $>1 \times 10^6$ $\log_{10}$ genome equivalents/ml or carriage of serotypes 1 or 5)

6) Definite pneumococcal pneumonia (*1*):

Pneumonia with a positive blood or pleural fluid culture.

Sample collection and laboratory procedures

We adhered to the WHO recommended methods for nasopharyngeal sample collection, handling and transport (*8*). A flocked, nylon swab was placed in 1 ml skim milk tryptone glucose glycerol media (STGG) immediately following collection. Swabs were stored in a fridge and transported to the National Center for Communicable Diseases where they were aliquoted and stored at ultra-low temperature within 8 hours of collection. Samples were shipped to the Murdoch Children's Research Institute (Parkville, Australia) on dry ice and stored at ultra-low temperature until testing. Nasopharyngeal swabs were tested for pneumococci using *lytA* real-time quantitative PCR (qPCR) and samples that were *lytA* qPCR positive (Ct value < 35) or equivocal (Ct value 35–40) were cultured on horse blood agar containing 5 µg/ml of gentamicin

(Oxoid) (*9*). DNA was extracted from the harvested α-haemolytic growth (*10*) followed by molecular serotyping by DNA microarray as previously described (*11*; C. von Mollendorf, unpub. data, https://doi.org/10.2139/ssrn.4488943). Microarray was performed using Senti-SPv1.5 microarrays (BUGS Bioscience) and analysed using Senti-NET, a custom web-based software (BUGS Bioscience). A total of 1000 cases per year were tested for pneumococci, including all cases with PEP (as this was the primary objective), and a random sample of remaining severe and non-severe cases.

Statistical analysis

To control for seasonal and long-term patterns we included an indicator variable for each elapsed calendar month (time elapsed) over the study period in the main model. We also explored three other options: fitting a spline function of time, Fourier terms and calendar month with a continuous time variable. The time elapsed variable was selected to control for seasonality as it resulted in improved model fit as measured by the Akaike's Information Criterion (AIC). No indicator variables were used to adjust for the impacts of the COVID-19 pandemic, as schools were closed from February 2020 to the end of the surveillance period with no significant reopening. We controlled for the impact of the COVID-19 pandemic by restricting to the pre-pandemic period (April 2015-Feb 2020) and then comparing results from the restricted model to a model including the total period (April 2015-June 2021). Model fit for all final models were evaluated using the AIC.

References

1. La Vincente SF, von Mollendorf C, Ulziibayar M, Satzke C, Dashtseren L, Fox KK, et al. Evaluation of a phased pneumococcal conjugate vaccine introduction in Mongolia using enhanced pneumonia surveillance and community carriage surveys: a study protocol for a prospective observational study and lessons learned. BMC Public Health. 2019;19:333. PubMed https://doi.org/10.1186/s12889-019-6639-y

2. Tsevelvaanchig U, Gouda H, Baker P, Hill PS. Role of emerging private hospitals in a post-Soviet mixed health system: a mixed methods comparative study of private and public hospital inpatient care in Mongolia. Health Policy Plan. 2017;32:476–86. PubMed

3. Center for Health Development, World Health Organization. Mongolia Health Indicators 2020. [cited 13 October 2023]. http://hdc.gov.mn/media/uploads/2022-05/health_indicator_2020_ENG.pdf

4. Cherian T, Mulholland EK, Carlin JB, Ostensen H, Amin R, de Campo M, et al. Standardized interpretation of paediatric chest radiographs for the diagnosis of pneumonia in epidemiological studies. Bull World Health Organ. 2005;83:353–9. PubMed

5. World Health Organization. IMCI integrated management of childhood illness. 2005 [cited 2022 Jun 16]. https://apps.who.int/iris/bitstream/handle/10665/42939/9241546441.pdf;jsessionid=02A6FF9DE8E4AF56D94A77AE0B9DEF51?sequence=1

6. von Mollendorf C, La Vincente S, Ulziibayar M, Suuri B, Luvsantseren D, Narangerel D, et al. Epidemiology of pneumonia in the pre-pneumococcal conjugate vaccine era in children 2-59 months of age, in Ulaanbaatar, Mongolia, 2015-2016. PLoS One. 2019;14:e0222423. PubMed https://doi.org/10.1371/journal.pone.0222423

7. Madhi SA, Klugman KP. World Health Organisation definition of "radiologically-confirmed pneumonia" may under-estimate the true public health value of conjugate pneumococcal vaccines. Vaccine. 2007;25:2413–9. PubMed https://doi.org/10.1016/j.vaccine.2006.09.010

8. Satzke C, Turner P, Virolainen-Julkunen A, Adrian PV, Antonio M, Hare KM, et al. Standard method for detecting upper respiratory carriage of *Streptococcus pneumoniae*: Updated recommendations from the World Health Organization Pneumococcal Carriage Working Group. Vaccine. 2013 2013/12/17/;32(1):165-79.

9. Carvalho MG, Tondella ML, McCaustland K, Weidlich L, McGee L, Mayer LW, et al. Evaluation and improvement of real-time PCR assays targeting lytA, ply, and psaA genes for detection of pneumococcal DNA. J Clin Microbiol. 2007;45:2460–6. PubMed https://doi.org/10.1128/JCM.02498-06

10. Satzke C, Dunne EM, Choummanivong M, Ortika BD, Neal EFG, Pell CL, et al. Pneumococcal carriage in vaccine-eligible children and unvaccinated infants in Lao PDR two years following the introduction of the 13-valent pneumococcal conjugate vaccine. Vaccine. 2019;37:296–305. PubMed https://doi.org/10.1016/j.vaccine.2018.10.077

11. Satzke C, Dunne EM, Porter BD, Klugman KP, Mulholland EK; PneuCarriage project group. The PneuCarriage Project: A Multi-Centre Comparative Study to Identify the Best Serotyping Methods for Examining Pneumococcal Carriage in Vaccine Evaluation Studies. [discussion e.]. PLoS Med. 2015;12:e1001903. PubMed https://doi.org/10.1371/journal.pmed.1001903

Appendix Table 1. Characteristics of 17,607 children 2–59 mo of age enrolled in pneumonia surveillance project from 4 study districts in Ulaanbaatar, Mongolia, April 2015 –June 2021

Category	Sub-category	Bayanzurkh (N = 4976) n/N (%)	Chingeltei (N = 4170) n/N (%)	Songinokhairkhan (N = 5332) n/N (%)	Sukhbaatar (N = 3129) n/N (%)	Total (N = 17,607) n/N (%)
Demographics						
Age group	2–23 mo	3563 (72)	3015 (72)	3805 (71)	2162 (69)	12545 (71)
	24–59 mo	1413 (28)	1155 (28)	1527 (29)	967 (31)	5062 (29)
Sex	Male	2747 (55)	2293 (55)	2806 (53)	1687 (54)	9533 (54)
Primary caregiver	Parent*	3764/4217 (89)	3421/3715 (92)	4148/4572 (91)	2371/2736 (87)	13704/15240 (90)
	Other relative	406/4217 (10)	277/3715 (7)	390/4572 (8)	184/2736 (7)	125715240 (8)
	Other	47/4217 (1)	17/3715 (1)	34/4572 (1)	181/2736 (7)	279/15240 (2)
Risk factors						
Seasons	Summer	365 (7)	334 (8)	457 (9)	310 (10)	1466 (8)
	Autumn	741 (15)	470 (11)	617 (12)	429 (14)	2257 (13)
	Winter	2739 (55)	2443 (59)	2964 (56)	1621 (52)	9767 (56)
	Spring	1131 (23)	923 (22)	1294 (24)	769 (25)	4117 (23)
Malnutrition†	Yes	249/4835 (5)	182/4137 (4)	341/5258 (6)	108/3069 (3)	880/17299 (5)
Currently breastfed	Yes	2326/4220 (55)	2172/3721 (58)	2672/4572 (58)	1452/2739 (53)	8622/15252 (56)
Caesarean section	Yes	990/4210 (23)	1073/3704 (29)	1011/4560 (22)	657/2736 (24)	3731/15210 (24)
Asthma	Yes	284/4148 (7)	353/3696 (10)	351/4540 (8)	199/2721 (7)	1187/15105 (8)
Children aged <5 y in the household	1 child	2961/4208 (70)	2579/3554 (73)	3060/4553 (67)	1822/2721 (67)	10422/15036 (69)
	≥2 children	1247/4208 (30)	975/3554 (27)	1493/4553 (33)	899/2721 (33)	4614/15036 (31)
Child attends daycare /kindergarten‡	Yes	933/4219 (22)	694/3717 (19)	915/4569 (20)	649/2739 (24)	3191/15244 (21)
Chimney in the home	Yes	2098/4215 (50)	2942/3710 (79)	3199/4569 (70)	1493/2739 (54)	9732/15233 (64)
Smoker in the home	Yes	1984/4216 (47)	1678/3712 (45)	2055/4571 (45)	1154/2738 (42)	6871/15237 (45)
Smokes inside the house	Yes	529/4201 (13)	314/3710 (8)	548/4565 (12)	261/2738 (9)	1,652/15214 (11)
Caregiver smokes	Yes	216/4206 (5)	178/3713 (5)	184/4569 (4)	134/2739 (5)	712/15227 (5)
Previous admission	Yes	1767/4199 (42)	1950/3693 (53)	2001/4548 (44)	1007/2713 (37)	6725/15153 (44)
Socioeconomic factors						
Fuel used in the home	Electricity or Gas	2085/4208 (49)	719/3703 (19)	1309/4568 (29)	1250/2735 (46)	5363/15214 (35)
	Coal or Wood	2123/4208 (51)	2984/3703 (81)	3259/4568 (71)	1485/2735 (54)	9851/15214 (65)
Housing[#]	Formal	2866/4219 (68)	2277/3716 (61)	2493/4571 (55)	1987/2739 (73)	9623/15245 (63)
	Informal	1353/4219 (32)	1439/3716 (39)	2078/4571 (45)	752/2739 (27)	5622/15245 (37)
Mother's education	Primary/Secondary	1956/4196 (47)	2143/3690 (58)	2647/4555 (58)	978/2734 (36)	7724/15175 (51)
	Tertiary	2240/4196 (53)	1547/3690 (42)	1908/4555 (42)	1756/2734 (64)	7451/15175 (49)
Income level§	Above minimum income	2514/4034 (62)	2284/3278 (70)	2313/4442 (52)	1504/2570 (58)	8615/14324 (60)
	At or below minimum income	1520/4034 (38)	994/3278 (30)	2129/4442 (48)	1066/2570 (42)	5709/14324 (40)
Crowding (people per room)	≤3	3139/4167 (75)	2486/3668 (68)	2890/4506 (64)	2203/2724 (81)	10718/15065 (71)
	>3	1028/4167 (25)	1182/3668 (32)	1616/4506 (36)	521/2724 (19)	4347/15065 (29)
Vaccination status						
PCV13 status¶	Pre-PCV13 period	2494/4722 (53)	2236/3804 (59)	1369/5096 (27)	882/2998 (29)	6981/16620 (42)
	Undervaccinated	1182/4722 (25)	1049/3804 (27)	1583/5096 (31)	1098/2998 (37)	4912/16620 (30)
	Vaccinated	1046/4722 (22)	519/3804 (14)	2144/5096 (42)	1018/2998 (34)	4727/16620 (28)
Severity of disease						
Length of hospital stay	≤7 d	3518/4975 (71)	3039/4169 (73)	4522/5329 (85)	2493 (80)	13572/17602 (77)
	8–14 d	1334/4975 (27)	1069/4169 (26)	691/5329 (13)	599 (19)	3693/17602 (21)

Category	Sub-category	Bayanzurkh (N = 4976) n/N (%)	Chingeltei (N = 4170) n/N (%)	Songinokhairkhan (N = 5332) n/N (%)	Sukhbaatar (N = 3129) n/N (%)	Total (N = 17,607) n/N (%)
	≥15 d	123/4975 (2)	61/4169 (1)	116/5329 (2)	37 (1)	337/17602 (2)
Outcome##	Died	14/4790 (0.3)	7/3923 (0.2)	12/4868 (0.2)	7/3038 (0.2)	40/16619 (0.2)
Hypoxic‖	Yes	1116/4734 (24)	790/4025 (20)	768/5140 (15)	518/2990 (17)	3192/16889 (19)
Primary endpoint pneumonia**	Yes	366/3609 (10)	395/3464 (11)	805/4367 (18)	247/2315 (11)	1813/13755 (13)
Severe pneumonia***	Yes	3718/4942 (75)	3270/4117 (79)	4271/5256 (81)	2208/3091 (71)	13467/17406 (77)
Very severe pneumonia^	Yes	2123/4942 (43)	1913/4117 (46)	1433/5256 (27)	966/3091 (31)	6434/17406 (37)
Probable pneumococcal pneumonia^^	Yes	309/3434 (9)	347/3434 (10)	549/4417 (12)	244/2317 (10)	1449/13602 (11)

*Mostly mothers (97%).
†Weight for age −2 standard deviations.
‡Kindergarten for children 2–5 y of age, daycare for children <2 y.
#Formal housing (houses and apartments) and informal housing (ger dwellings).
§Minimum income was considered 170,000₮ per person/per month.
¶Children were considered PCV13 vaccinated if they have received at least two doses when administered at less than 12 mo of age or at least one dose when administered at greater than or equal to 12 mo of age.
##Number of children who died during hospital stay.
‖Hypoxic defined as an oxygen saturation <90%.
**WHO defined primary endpoint pneumonia.
***Severe pneumonia defined according to WHO integrated management of childhood illness 2005 case definition.
^Very severe pneumonia included severe cases complicated by empyema, intensive care unit admission, persistent severe disease post-discharge, hypoxia or death.
^^Probable pneumococcal pneumonia was defined as elevated C-reactive protein with either PEP or high pneumococcal nasopharyngeal carriage (either high density carriage of any serotype greater than 1 × $\log_{10}$ GE/mL, or any carriage of serotypes 1 or 5).

Appendix Table 2. Characteristics of children 2–59 months of age hospitalised with clinical pneumonia in pre- and post-PCV period, April 2015 to June 2021

Category	Sub-category	Pre-PCV period (N=7304) n (%)	Post-PCV period (N=10303) n (%)	p-value*
Demographics				
Age group	2-23 months	5196 (71)	7349 (71)	0.78
	24-59 months	2108 (29)	2954 (29)	
Sex	Male	3958 (54)	5575 (54)	0.91
District	Bayanzurkh	2654 (36)	2322 (22)	<0.001
	Chingeltei	2309 (32)	1860 (18)	
	Songinokhairkhan	1400 (19)	3932 (38)	
	Sukhbaatar	941 (13)	2189 (21)	
Primary caregiver	Parent	5256/5836 (90)	8448/9404 (90)	<0.001
	Other relative	502/5836 (10)	755/9404 (8)	
	Other	78/5836 (1)	201/9404 (2)	
Risk factors				
Seasons	Summer	608 (8)	858 (8)	<0.001
	Autumn	942 (13)	1316 (13)	
	Winter	3731 (51)	6036 (59)	
	Spring	2024 (28)	2093 (20)	
Malnutrition†	Yes	345/7128 (5)	535/10171 (5)	0.21
Currently breastfed	Yes	3226/5837 (55)	5396/9415 (57)	0.01
Caesarean	Yes	1489/5818 (26)	2242/9392 (24)	0.01
Asthma	Yes	574/5760 (10)	613/9345 (6)	<0.001
Children aged <5 years in the household	1 child	4012/5726 (70)	6410/9310 (69)	0.12
	≥2 children	1714/5726 (30)	2900/9310 (31)	
Child attends daycare /kindergarten‡	Yes	1256/5821 (22)	1935/9398 (21)	0.15
Chimney in the home	Yes	3752/5830 (64)	5980/9403 (64)	0.34
Smoker in the home	Yes	2582/5831 (44)	4289/9406 (46)	0.12
Smokes inside the house	Yes	668/5812 (11)	984/9402 (10)	0.05
Caregiver smokes	Yes	310/5824 (5)	402/9403 (4)	0.003
Previous admission	Yes	2782/5794 (48)	3943/9359 (42)	<0.001
Socioeconomic factors				
Fuel used in the home	Electricity or Gas	1987/5828 (34)	3376/9386 (36)	0.02
	Coal or Wood	3841/5828 (66)	6010/9386 (64)	

Category	Sub-category	Pre-PCV period (N=7304) n (%)	Post-PCV period (N=10303) n (%)	p-value*
Housing	Formal	3657/5837 (63)	5966/9408 (63)	0.34
	Informal	2180/5837 (37)	3442/9408 (37)	
Mother's education	Primary/Secondary	2938/5793 (51)	4786/9382 (51)	0.72
	Tertiary	2855/5793 (49)	4596/9382 (49)	
Income level§	Above minimum income	3449/5422 (64)	5166/8902 (58)	<0.001
	At or below minimum income	1973/5422 (36)	3736/8902 (42)	
Crowding (people per room)	<=3	4096/5732 (71)	6622/9333 (71)	0.52
	>3	1636/5732 (29)	2711/9333 (29)	
Severity of disease				
Length of hospital stay	<=7 days	5518/7299 (76)	8053 (78)	<0.001
	8-14 days	1638/7299 (22)	2055 (20)	
	>=15 days	143/7299 (2)	195 (2)	
Outcome	Died	18/6345 (0.3)	22/10274 (0.2)	0.37
Hypoxic‖	Yes	1460/6737 (22)	1732/10153 (17)	<0.001
Primary endpoint pneumonia**	Yes	739/5213 (14)	1074/8542 (13)	0.007
Severe pneumonia***	Yes	5676/7146 (79)	7791/10260 (76)	<0.001
Very severe pneumonia^	Yes	2751/7146 (38)	3683/10260 (36)	0.009
Probable pneumococcal pneumonia^^	Yes	695/4390 (14)	754/7763 (9)	<0.001

*p-values compared pre- versus post-PCV period using chi-squared test.
†Weight for age -2 standard deviations.
‡Kindergarten for children 2-5 years of age, daycare for children <2 years.
§Minimum income was considered 170,000₮ per person/per month.
‖Hypoxic defined as an oxygen saturation <90%.
**WHO defined primary end point pneumonia.
***Severe pneumonia defined according to WHO integrated management of childhood illness 2005 case definition.
^Very severe pneumonia included severe cases complicated by empyema, intensive care unit admission, persistent severe disease post-discharge. hypoxia or death.
^^Probable pneumococcal pneumonia was defined as elevated C-reactive protein with either PEP or high pneumococcal nasopharyngeal carriage (either high density carriage of any serotype greater than $1 \times \log_{10}$ GE/mL, or any carriage of serotypes 1 or 5).

Appendix Table 3. Crude incidence and incidence rate ratios for hospitalised pneumonia by district and diagnosis in the pre- and post-PCV period, for children 2-59 months, from April 2015 to March 2020 and April 2015 to March 2021

Variable	District	Incidence rate pre-PCV introduction (per 1000 pop)	Incidence rate post-PCV introduction until March 2020 (per 1000 pop)*	Incidence rate ratio (95% confidence interval) comparing pre-PCV to post-PCV until March 2020*	Incidence rate post-PCV introduction until March 2021 (per 1000 pop)†	Incidence rate ratio (95% confidence interval) comparing pre-PCV to post-PCV until March 2021†
All clinical pneumonia	All	33.9 (33.2-34.7)	26.9 (26.4-27.5)	0.79 (0.77-0.82)	21.3 (20.9-21.8)	0.63 (0.61-0.65)
	BZD	28.7 (27.6-29.8)	19.1 (18.4-20.0)	0.67 (0.63-0.71)	14.7 (14.1-15.3)	0.51 (0.48-0.54)
	CHD	41.9 (40.2-43.7)	47.4 (45.2-49.6)	1.13 (1.06-1.20)	34.7 (33.1-36.3)	0.83 (0.78-0.88)
	SKD	29.2 (27.7-30.8)	25.1 (24.3-25.9)	0.86 (0.81-0.91)	20.5 (19.9-21.2)	0.70 (0.66-0.75)
	SBD	48.0 (45.0-51.1)	33.7 (32.3-35.2)	0.70 (0.65-0.76)	27.9 (26.7-29.1)	0.58 (0.54-0.63)
Primary endpoint pneumonia‡	All	3.5 (3.2-3.7)	2.8 (2.6-2.9)	0.80 (0.73-0.88)	2.2 (2.1-2.4)	0.64 (0.58-0.71)
	BZD	2.1 (1.8-2.4)	1.3 (1.1-1.5)	0.63 (0.50-0.78)	1.1 (0.9-1.2)	0.51 (0.41-0.63)
	CHD	4.4 (3.9-5.0)	3.8 (3.2-4.5)	0.86 (0.69-1.05)	2.9 (2.4-3.4)	0.65 (0.53-0.80)
	SKD	4.2 (3.7-4.9)	3.9 (3.6-4.2)	0.91 (0.78-1.08)	3.1 (2.9-3.4)	0.74 (0.63-0.87)
	SBD	5.1 (4.2-6.2)	2.2 (1.9-2.6)	0.43 (0.33-0.56)	1.8 (1.5-2.2)	0.36 (0.28-0.47)
Severe pneumonia§	All	26.4 (25.7-27.1)	20.4 (20.0-20.9)	0.77 (0.75-0.80)	16.1 (15.8-16.5)	0.61 (0.59-0.63)
	BZD	22.4 (21.5-23.4)	13.6 (13.0-14.3)	0.61 (0.57-0.65)	10.4 (9.9-10.9)	0.46 (0.43-0.49)
	CHD	32.2 (30.7-33.7)	38.1 (36.1-40.1)	1.18 (1.10-1.27)	27.9 (26.5-29.3)	0.86 (0.81-0.93)
	SKD	23.2 (21.8-24.6)	20.3 (19.6-21.0)	0.88 (0.82-0.94)	16.5 (15.9-17.1)	0.71 (0.66-0.76)
	SBD	36.5 (33.9-39.3)	22.9 (21.7-24.1)	0.63 (0.57-0.69)	19.0 (18.0-20.0)	0.52 (0.47-0.57)
Very severe pneumonia¶	All	12.8 (12.3-13.3)	9.5 (9.2-9.8)	0.74 (0.71-0.78)	7.6 (7.4-7.9)	0.59 (0.57-0.62)
	BZD	12.5 (11.8-13.2)	7.9 (7.4-8.4)	0.63 (0.58-0.69)	6.1 (5.7-6.5)	0.49 (0.45-0.53)
	CHD	17.4 (16.3-18.6)	23.8 (22.2-25.5)	1.37 (1.25-1.50)	17.6 (16.6-18.8)	1.01 (0.93-1.11)
	SKD	9.0 (8.2-10.0)	6.4 (6.0-6.8)	0.71 (0.63-0.80)	5.2 (4.9-5.5)	0.58 (0.52-0.65)
	SBD	10.5 (9.1-12.0)	11.6 (10.8-12.5)	1.11 (0.95-1.30)	9.7 (9.0-10.4)	0.92 (0.79-1.08)
Hypoxic pneumonia‖	All	6.8 (6.4-7.2)	4.5 (4.3-4.7)	0.66 (0.61-0.71)	3.6 (3.4-3.8)	0.53 (0.49-0.56)
	BZD	5.9 (5.5-6.5)	4.5 (4.2-4.9)	0.76 (0.68-0.86)	3.5 (3.3-3.9)	0.60 (0.53-0.67)
	CHD	8.9 (8.2-9.8)	7.5 (6.7-8.5)	0.84 (0.72-0.98)	5.5 (4.9-6.2)	0.62 (0.53-0.71)
	SKD	5.2 (4.6-5.9)	3.3 (3.0-3.6)	0.63 (0.54-0.73)	2.7 (2.5-2.9)	0.51 (0.44-0.60)
	SBD	8.5 (7.2-9.8)	5.4 (4.8-6.0)	0.63 (0.53-0.77)	4.5 (4.0-5.0)	0.53 (0.44-0.64)
Probable pneumococcal pneumonia**	All	3.2 (3.0-3.5)	2.0 (1.8-2.1)	0.62 (0.56-0.69)	1.5 (1.4-1.7)	0.48 (0.43-0.53)
	BZD	2.1 (1.8-2.4)	1.0 (0.8-1.2)	0.48 (0.38-0.61)	0.7 (0.6-0.9)	0.36 (0.28-0.45)
	CHD	4.3 (3.8-4.9)	2.8 (2.3-3.4)	0.66 (0.52-0.84)	2.0 (1.6-2.4)	0.46 (0.36-0.58)
	SKD	3.5 (3.0-4.0)	2.4 (2.2-2.7)	0.70 (0.58-0.85)	2.0 (1.8-2.2)	0.57 (0.47-0.68)
	SBD	4.9 (4.0-6.0)	2.3 (1.9-2.7)	0.46 (0.35-0.60)	1.8 (1.5-2.2)	0.37 (0.29-0.49)

BZD = Bayanzurkh District, CHD = Chingeltei District, SKD = Songinokhairkhan District, SBD = Sukhbaatar District.
Annual incidence rates calculated from April to March. * Incidence rate and incidence rate ratio up to March 2020, approximately start of COVID pandemic impact on the surveillance programme.
†Incidence rate and incidence rate ratio up to March 2021, near study completion.
‡WHO defined primary end point pneumonia.
§Severe pneumonia defined according to WHO integrated management of childhood illness 2005 case definition.
¶Very severe pneumonia included severe cases complicated by empyema, intensive care unit admission, persistent severe disease post-discharge, hypoxia or death.
‖Hypoxic pneumonia defined as an oxygen saturation <90%.
**Probable pneumococcal pneumonia was defined as elevated C-reactive protein with either PEP or high pneumococcal nasopharyngeal carriage (either high density carriage of any serotype greater than 1 × $\log_{10}$ GE/mL, or any carriage of serotypes 1 or 5).

Appendix Table 4. Crude incidence of hospitalised clinical pneumonia by age group and diagnosis in the pre- and post-PCV period, from April 2015 to March 2020 and April 2015 to March 2021.

**Variable**	**Age group**	**Incidence rate pre-PCV introduction (per 1000 pop)**	**Incidence rate post-PCV introduction (per 1000 pop) until March 2020***	Incidence rate ratio **(95% confidence interval) comparing pre-PCV to post-PCV until March 2020***	**Incidence rate post-PCV introduction (per 1000 pop) until March 2021†**	Incidence rate ratio **(95% confidence interval) comparing pre-PCV to post-PCV until March 2021†**
All clinical pneumonia	2-23 months	63.2 (61.5-64.9)	53.7 (52.5-55.0)	0.85 (0.82-0.88)	42.2 (41.3-43.2)	0.67 (0.64-0.69)
	24-59 months	15.8 (15.2-16.5)	12.1 (11.6-12.5)	0.76 (0.72-0.80)	9.6 (9.2-9.9)	0.60 (0.57-0.64)
	2-59 months	33.9 (33.2-34.7)	26.9 (26.4-27.5)	0.79 (0.77-0.82)	21.3 (20.9-21.8)	0.63 (0.61-0.65)
Primary endpoint pneumonia‡	2-23 months	6.4 (5.8-6.9)	5.7 (5.3-6.1)	0.89 (0.80-1.00)	4.5 (4.2-4.8)	0.71 (0.64-0.80)
	24-59 months	1.6 (1.4-1.8)	1.2 (1.0-1.3)	0.72 (0.60-0.86)	0.9 (0.8-1.0)	0.57 (0.47-0.68)
	2-59 months	3.5 (3.2-3.7)	2.8 (2.6-2.9)	0.80 (0.73-0.88)	2.2 (2.1-2.4)	0.64 (0.58-0.71)
Severe pneumonia§	2-23 months	48.7 (47.2-50.3)	40.4 (39.4-41.5)	0.83 (0.80-0.86)	31.7 (30.9-32.6)	0.65 (0.62-0.68)
	24-59 months	12.5 (11.9-13.1)	9.3 (8.9-9.7)	0.74 (0.70-0.79)	7.4 (7.1-7.7)	0.59 (0.55-0.63)
	2-59 months	26.4 (25.7-27.1)	20.4 (20.0-20.9)	0.77 (0.75-0.80)	16.1 (15.8-16.5)	0.61 (0.59-0.63)
Very severe pneumonia¶	2-23 months	23.0 (22.0-24.1)	19.4 (18.7-20.2)	0.84 (0.79-0.90)	15.4 (14.9-16.0)	0.67 (0.63-0.71)
	24-59 months	6.5 (6.0-6.9)	4.0 (3.7-4.3)	0.62 (0.56-0.68)	3.2 (3.0-3.4)	0.49 (0.45-0.54)
	2-59 months	12.8 (12.3-13.3)	9.5 (9.2-9.8)	0.74 (0.71-0.78)	7.6 (7.4-7.9)	0.59 (0.57-0.62)
Hypoxic pneumonia‖	2-23 months	12.2 (11.5-13.0)	9.1 (8.6-9.6)	0.74 (0.68-0.81)	7.2 (6.8-7.6)	0.59 (0.54-0.64)
	24-59 months	3.4 (3.1-3.7)	1.9 (1.7-2.1)	0.56 (0.49-0.64)	1.5 (1.4-1.7)	0.45 (0.39-0.51)
	2-59 months	6.8 (6.4-7.2)	4.5 (4.3-4.7)	0.66 (0.61-0.71)	3.6 (3.4-3.8)	0.53 (0.49-0.57)
Probable pneumococcal pneumonia**	2-23 months	5.5 (5.0-6.0)	3.5 (3.2-3.8)	0.63 (0.55-0.72)	2.7 (2.4-2.9)	0.49 (0.43-0.56)
	24-59 months	1.8 (1.6-2.0)	1.2 (1.0-1.3)	0.65 (0.55-0.78)	0.9 (0.8-1.0)	0.51 (0.42-0.60)
	2-59 months	3.2 (3.0-3.5)	2.0 (1.8-2.1)	0.62 (0.56-0.69)	3.2 (3.0-3.5)	0.48 (0.43-0.53)

Annual incidence rates calculated from April to March.
*Incidence rate and incidence rate ratio up to March 2020, approximately start of COVID pandemic impact on the surveillance programme.
†Incidence rate and incidence rate ratio up to March 2021, near study completion.
‡WHO defined primary end point pneumonia.
§Severe pneumonia defined according to WHO integrated management of childhood illness 2005 case definition.
¶Very severe pneumonia included severe cases complicated by empyema, intensive care unit admission, persistent severe disease post-discharge, hypoxia or death.
‖Hypoxic pneumonia defined as an oxygen saturation <90%.
**Probable pneumococcal pneumonia was defined as elevated C-reactive protein with either PEP or high pneumococcal nasopharyngeal carriage (either high density carriage of any serotype greater than 1 × $\log_{10}$ GE/mL, or any carriage of serotypes 1 or 5).

Appendix Table 5. Crude incidence rates by year for hospitalised clinical pneumonia by district and diagnosis for children aged 2-59 months, April 2015 to June 2021

		Crude Incidence rates (per 1000 population)					
**District**	**Year**	**All clinical pneumonia**	**Primary endpoint pneumonia***	**Severe pneumonia‡**	**Very severe pneumonia§**	**Hypoxic pneumonia¶**	**Probable pneumococcal pneumonia[‖]**
**Bayanzurkh District**	2015	17.11	1.28	14.48	6.45	3.28	1.16
	2016	35.94	2.42	28.56	17.24	8.08	2.54
	2017	22.97	1.74	14.98	8.41	4.34	1.81
	2018	24.39	1.83	17.83	10.41	5.49	1.40
	2019	14.35	0.78	10.30	6.13	3.76	0.51
	2020	5.36	0.68	3.65	2.65	1.85	0.05
	2021	0.43	0.14	0.26	0.21	0.19	0.07
**Chingeltei District**	2015	13.12	2.04	8.22	5.16	2.50	1.74
	2016	45.61	4.21	34.30	18.26	9.79	4.58
	2017	51.16	6.01	40.98	20.35	11.51	5.62
	2018	55.21	4.62	45.26	27.55	10.12	4.12
	2019	46.00	3.40	36.62	23.38	6.45	2.58
	2020	19.18	1.70	15.47	11.17	2.97	0.36
	2021	1.26	0.13	1.07	0.95	0.32	0.00
**Songinokhairkhan District**	2015	15.26	3.07	10.99	4.53	2.56	1.73
	2016	36.57	5.05	30.56	9.96	5.47	4.26
	2017	24.96	3.72	20.48	5.70	3.51	3.12
	2018	26.42	3.59	21.82	7.45	3.84	2.78
	2019	22.57	3.42	17.46	6.21	2.92	1.30
	2020	7.41	1.30	5.42	1.81	0.79	0.56
	2021	0.48	0.03	0.34	0.21	0.16	0.03
**Sukhbaatar District**	2015	25.06	2.38	18.17	3.62	2.02	1.66
	2016	63.71	5.83	46.52	21.22	14.91	5.83
	2017	44.66	2.78	29.53	9.38	6.78	3.87
	2018	31.48	1.99	20.73	11.54	4.71	2.42
	2019	16.55	1.40	12.73	9.55	1.65	0.64
	2020	6.76	0.46	5.18	3.15	1.12	0.33
	2021	1.72	0.14	1.17	0.48	0.21	0.00

*WHO defined primary end point pneumonia.
‡Severe pneumonia defined according to WHO integrated management of childhood illness 2005 case definition.
§Very severe pneumonia included severe cases complicated by empyema, intensive care unit admission, persistent severe disease post-discharge, hypoxia or death.
¶Hypoxic pneumonia defined as an oxygen saturation <90%.
‖Probable pneumococcal pneumonia included PEP or high pneumococcal nasopharyngeal carriage with a C-reactive protein ≥40 mg/dL.

Appendix Table 6. Adjusted incidence rate ratios (aIRR) with 95% confidence interval (95%CI) for different pneumonia endpoints comparing the pre- and post-vaccine periods (April 2015 – Feb 2020 and April 2015 – June 2021) overall and separately in the four districts using negative binomial models

Variable	aIRR (95% CI) until Feb 2020	aIRR (95% CI) until June 2021
All clinical pneumonia		
All districts*	1.01 (0.87-1.17)	1.02 (0.88-1.18)
Bayanzurkh District‡	0.71 (0.59-0.85)	0.71 (0.59-0.84)
Chingeltei District‡	1.68 (1.41-2.01)	1.64 (1.38-1.94)
Songinokhairkhan District‡	0.86 (0.70-1.07)	0.85 (0.69-1.05)
Sukhbaatar District‡	0.64 (0.51-0.79)	0.65 (0.52-0.80)
Primary endpoint pneumonia§		
All districts*	0.72 (0.56-0.93)	0.76 (0.59-0.97)
Bayanzurkh District‡	0.68 (0.49-0.93)	0.73 (0.53-1.00)
Chingeltei District‡	1.01 (0.74-1.39)	1.05 (0.77-1.43)
Songinokhairkhan District‡	0.74 (0.53-1.04)	0.73 (0.52-1.02)
Sukhbaatar District‡	0.35 (0.24-0.51)	0.36 (0.24-0.53)
Severe pneumonia¶		
All districts*	0.97 (0.82-1.15)	1.00 (0.85-1.17)
Bayanzurkh District‡	0.61 (0.50-0.74)	0.60 (0.50-0.73)
Chingeltei District‡	1.72 (1.42-2.09)	1.72 (1.43-2.06)
Songinokhairkhan District‡	0.86 (0.68-1.08)	0.84 (0.67-1.05)
Sukhbaatar District‡	0.58 (0.45-0.73)	0.59 (0.47-0.75)
Very severe pneumonia		
All districts*	0.77 (0.64-0.93)	0.80 (0.66-0.96)
Bayanzurkh District‡	0.46 (0.37-0.57)	0.47 (0.38-0.58)
Chingeltei District‡	1.43 (1.15-1.77)	1.45 (1.18-1.77)
Songinokhairkhan District‡	0.47 (0.36-0.60)	0.46 (0.35-0.59)
Sukhbaatar District‡	0.71 (0.53-0.94)	0.71 (0.54-0.94)
Hypoxic pneumonia**		

Variable	aIRR (95% CI) until Feb 2020	aIRR (95% CI) until June 2021
All districts*	0.83 (0.67-1.04)	0.84 (0.68-1.04)
Bayanzurkh District‡	0.79 (0.60-1.04)	0.81 (0.62-1.06)
Chingeltei District‡	1.14 (0.86-1.51)	1.09 (0.84-1.43)
Songinokhairkhan District‡	0.60 (0.43-0.83)	0.59 (0.43-0.81)
Sukhbaatar District‡	0.64 (0.46-0.91)	0.65 (0.46-0.91)
Probable pneumococcal pneumonia***		
All districts*	0.77 (0.61-0.97)	0.75 (0.60-0.95)
Bayanzurkh District‡	0.65 (0.48-0.88)	0.64 (0.47-0.86)
Chingeltei District‡	1.24 (0.91-1.68)	1.16 (0.86-1.57)
Songinokhairkhan District‡	0.69 (0.51-0.93)	0.69 (0.51-0.93)
Sukhbaatar District‡	0.45 (0.32-0.65)	0.46 (0.32-0.65)

*Negative binomial model included time elapsed, age group, district and PCV13.
‡Negative binomial model included time elapsed, age group, district and PCV13 with interaction term between district and PCV13 for separate districts.
§WHO defined primary end point pneumonia.
¶Severe pneumonia defined according to WHO integrated management of childhood illness 2005 case definition.
||Very severe pneumonia included severe cases complicated by empyema, intensive care unit admiss.ion, persistent severe disease post-discharge, hypoxia or death.
**Hypoxic pneumonia defined as an oxygen saturation <90%.
***Probable pneumococcal pneumonia included PEP or high pneumococcal nasopharyngeal carriage with a C-reactive protein ≥40 mg/dL.

Appendix Table 7. Sensitivity analyses using negative binomial models for different pneumonia endpoints comparing the pre- and post-vaccine periods overall and separately in the four districts (April 2015 – June 2021).

Variable	aIRR (95% confidence interval) with delayed PCV period in children 2-59 months†	aIRR (95% confidence interval) in children 2-23 months‡	aIRR (95% confidence interval) in children 24-59 months‡
All clinical pneumonia			
All districts	0.76 (0.64-0.91)	1.09 (0.91-1.30)	0.88 (0.73-1.07)
Bayanzurkh district	0.61 (0.50-0.74)	0.77 (0.63-0.94)	0.63 (0.50-0.80)
Chingeltei district	1.37 (1.11-1.68)	1.76 (1.45-2.13)	1.38 (1.10-1.74)
Songinokhairkhan district	0.75 (0.62-0.91)	0.82 (0.65-1.04)	0.84 (0.64-1.11)
Sukhbaatar district	0.51 (0.42-0.62)	0.67 (0.53-0.86)	0.58 (0.44-0.78)
Primary endpoint pneumonia§			
All districts	0.74 (0.57-0.96)	0.88 (0.67-1.15)	0.62 (0.43-0.90)
Bayanzurkh district	0.72 (0.51-1.01)	0.88 (0.63-1.21)	0.54 (0.33-0.89)
Chingeltei district	1.04 (0.71-1.51)	1.29 (0.94-1.76)	0.73 (0.45-1.20)
Songinokhairkhan district	0.83 (0.62-1.10)	0.71 (0.51-0.99)	0.86 (0.52-1.43)
Sukhbaatar district	0.43 (0.30-0.62)	0.37 (0.24-0.56)	0.35 (0.20-0.61)
Severe pneumonia¶			
All districts	0.76 (0.62-0.92)	1.05 (0.87-1.28)	0.87 (0.70-1.09)
Bayanzurkh district	0.54 (0.44-0.68)	0.66 (0.54-0.81)	0.55 (0.42-0.72)
Chingeltei district	1.42 (1.14-1.79)	1.81 (1.48-2.21)	1.49 (1.15-1.94)
Songinokhairkhan district	0.73 (0.58-0.90)	0.80 (0.63-1.03)	0.84 (0.61-1.16)
Sukhbaatar district	0.50 (0.40-0.63)	0.61 (0.47-0.79)	0.53 (0.39-0.73)
Very severe pneumonia||			
All districts	0.70 (0.56-0.86)	0.95 (0.77-1.18)	0.61 (0.48-0.79)
Bayanzurkh district	0.46 (0.36-0.59)	0.58 (0.46-0.74)	0.35 (0.26-0.47)
Chingeltei district	1.31 (1.01-1.69)	1.71 (1.36-2.15)	1.08 (0.81-1.43)
Songinokhairkhan district	0.53 (0.41-0.67)	0.51 (0.38-0.68)	0.41 (0.28-0.59)
Sukhbaatar district	0.68 (0.52-0.88)	0.79 (0.58-1.08)	0.62 (0.41-0.93)
Hypoxic pneumonia**			
All districts	0.86 (0.69-1.08)	0.96 (0.75-1.22)	0.69 (0.52-0.93)
Bayanzurkh district	1.09 (0.82-1.43)	0.97 (0.73-1.29)	0.67 (0.46-0.96)
Chingeltei district	1.20 (0.87-1.66)	1.36 (1.02-1.82)	0.81 (0.55-1.19)
Songinokhairkhan district	0.83 (0.63-1.09)	0.61 (0.44-0.86)	0.54 (0.35-0.85)
Sukhbaatar district	0.54 (0.40-0.72)	0.61 (0.43-0.88)	0.69 (0.43-1.12)
Probable pneumococcal pneumonia***			
All districts	0.73 (0.57-0.94)	0.70 (0.53-0.92)	0.80 (0.57-1.13)
Bayanzurkh district	0.60 (0.40-0.88)	0.60 (0.42-0.86)	0.73 (0.46-1.18)
Chingeltei district	1.11 (0.70-1.76)	1.29 (0.90-1.85)	0.92 (0.57-1.50)
Songinokhairkhan district	0.84 (0.63-1.11)	0.57 (0.41-0.81)	0.94 (0.58-1.53)
Sukhbaatar district	0.56 (0.39-0.80)	0.37 (0.24-0.56)	0.57 (0.33-0.97)

†Negative binomial model included time elapsed, district and PCV13_delay with interaction term between district and PCV13_delay. PCV impact assumed from one-year post-PCV introduction.
‡Negative binomial model included time elapsed, season, district and PCV13 stratified by age group
§WHO defined primary end point pneumonia.
¶Severe pneumonia defined according to WHO integrated management of childhood illness 2005 case definition.
||Very severe pneumonia included severe cases complicated by empyema, intensive care unit admission, persistent severe disease post-discharge, hypoxia or death.
**Hypoxic pneumonia defined as an oxygen saturation <90%.
***Probable pneumococcal pne.umonia included PEP or high pneumococcal nasopharyngeal carriage with a C-reactive protein ≥40 mg/dL.

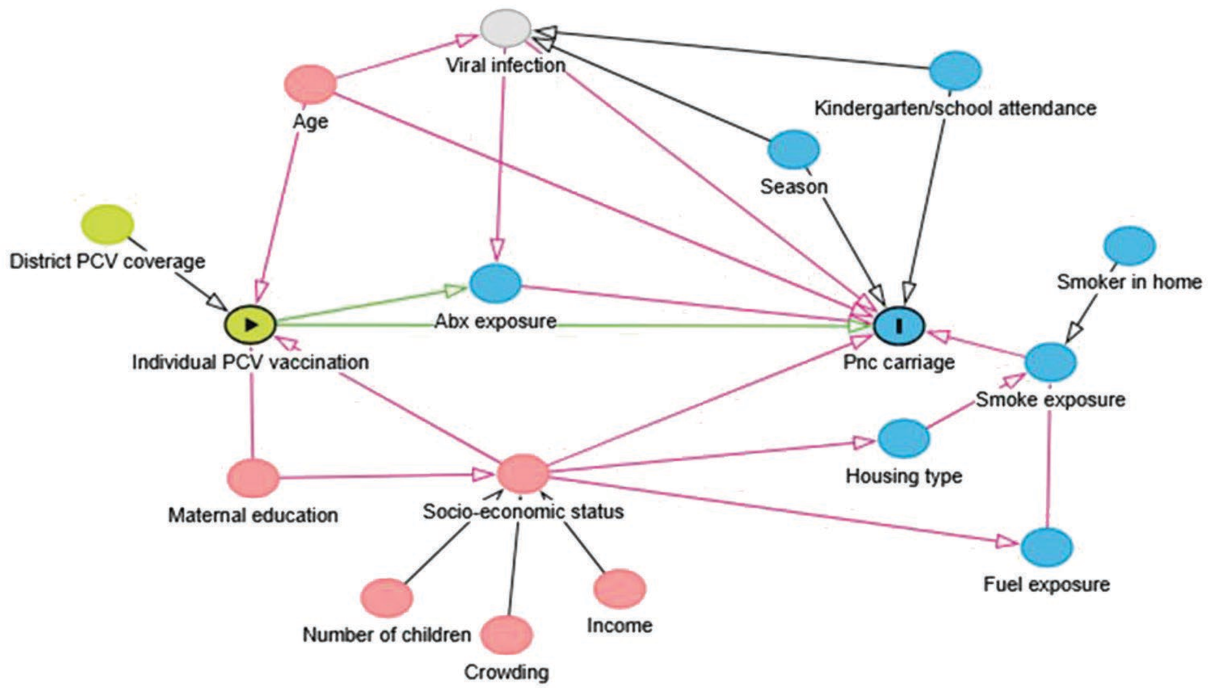

Appendix Figure 1. Directed acyclic graph (DAG) of the association between PCV13 vaccination (exposure) and pneumococcal carriage (outcome) The DAG was used to identify potential confounding variables. The green line highlights the causal relationship under investigation and the pink lines highlight potential biasing pathways. The blue variables are ancestors of the outcome, yellow variables ancestors of the exposure and red variables ancestors of both exposure and outcome. Grey variables represent unobserved variables. Based on this DAG, we identified that adjusting for age group, housing-type, maternal education, household income, household crowding, number of children under five years of age, household fuel type, season and antibiotic exposure may block biasing pathways.

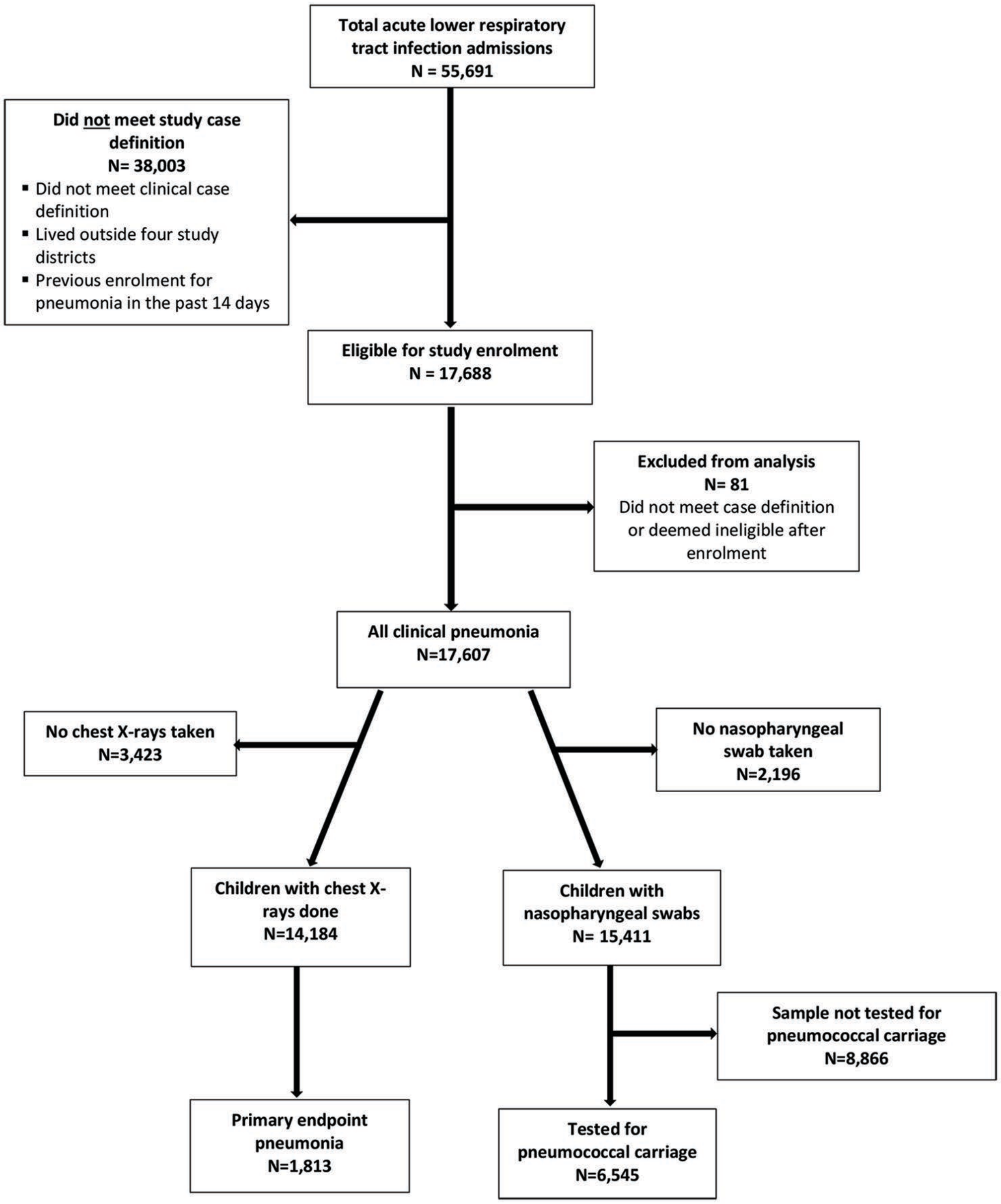

Appendix Figure 2. Flow chart of study participants with pneumonia admissions in four districts of Ulaanbaatar, Mongolia, April 2015–June 2021.

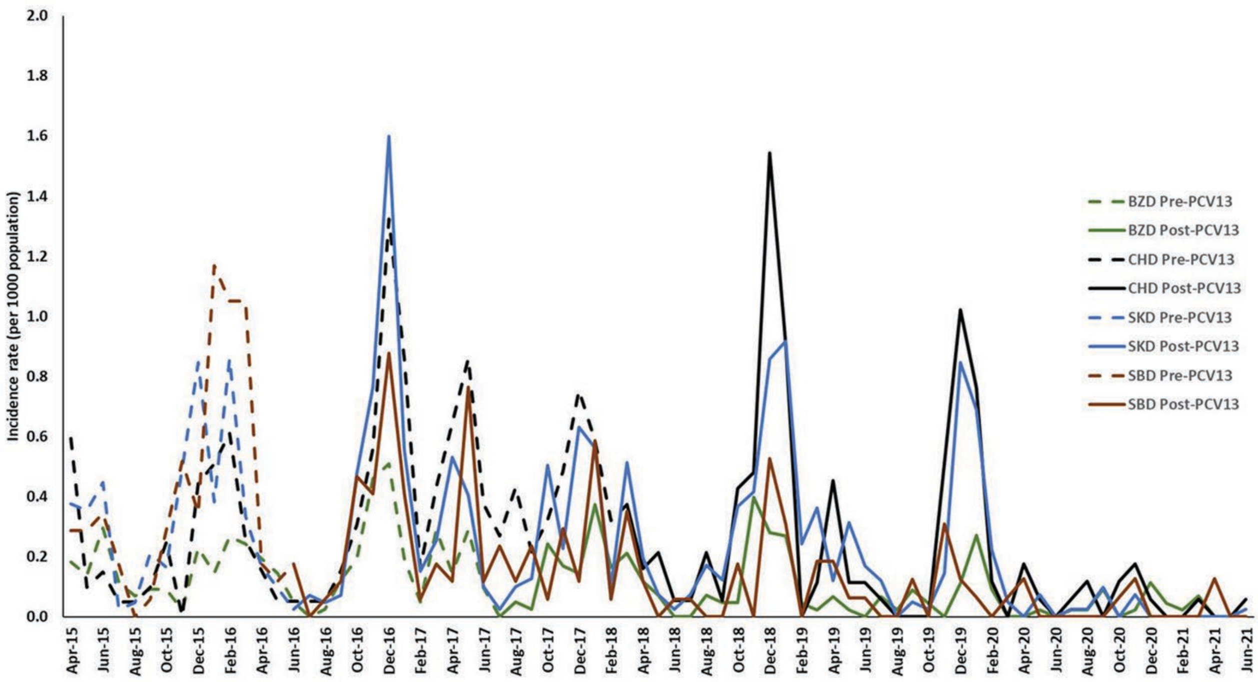

Appendix Figure 3. Primary endpoint pneumonia incidence rates by month and district in children 2-59 months of age, Ulaanbaatar, Mongolia, April 2015 – June 2021.

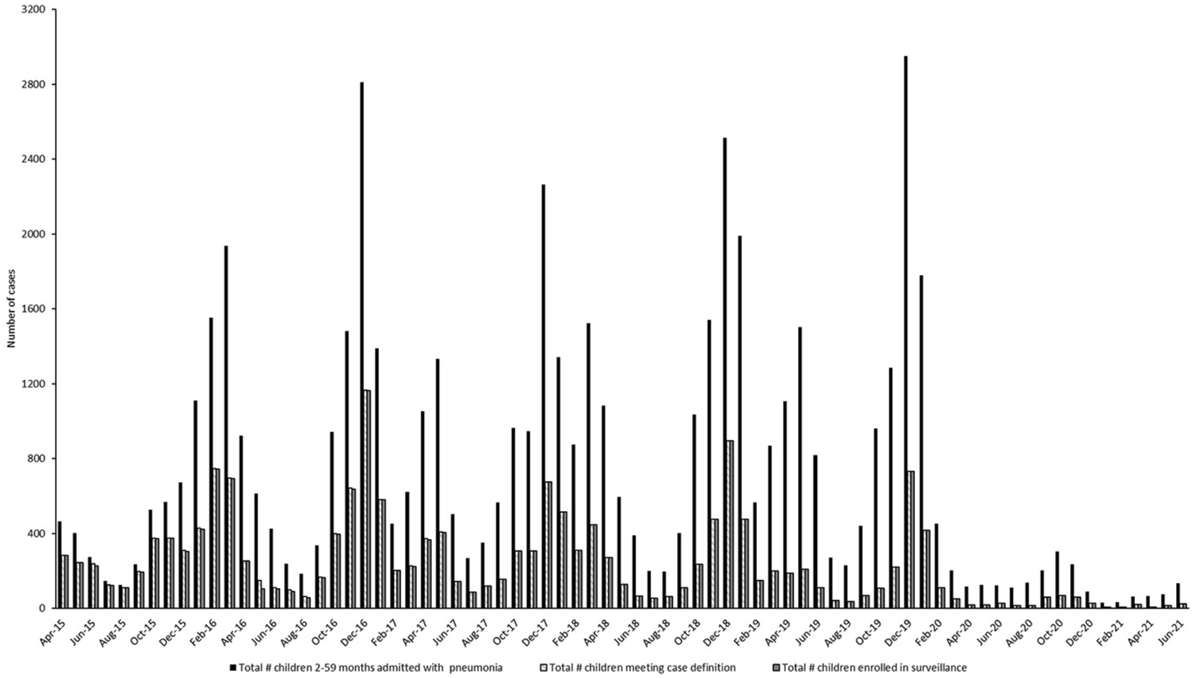

Appendix Figure 4. Total pneumonia cases admitted, meeting case definition and enrolled in pneumonia surveillance programme for children 2-59 months in all four districts, April 2015 to June 2021.
